# Effect of unprocessed red meat on obesity and related factors: A systematic review and meta‐analysis

**DOI:** 10.1002/oby.24322

**Published:** 2025-07-25

**Authors:** Md Akheruzzaman, Marleigh Hefner, Daniel Baller, Shane Clark, Zahra Feizy, Diana M. Thomas, Nikhil V. Dhurandhar

**Affiliations:** ^1^ Department of Nutritional Sciences Texas Tech University Lubbock Texas USA; ^2^ US Army Intelligence and Security Command Virginia USA; ^3^ Department of Mathematical Sciences United States Military Academy West Point New York USA; ^4^ Department of Mathematics Baruch College New York New York USA; ^5^ Department of Human Performance The University of Texas Permian Basin Odessa Texas USA

## Abstract

**Objective:**

The objective of this study was to conduct a systematic review and meta‐analysis of intervention trials that have determined the effect of unprocessed red meat (URM) intake on obesity‐related outcomes.

**Methods:**

The populations, interventions, controls, and outcomes (PICO) framework was used to create questions to search seven databases from July 29, 2020, to August 21, 2020. Two reviewers independently screened 5630 references. English‐language intervention trials in adults testing the effect of URM on obesity‐related outcomes were included. Twenty‐four studies met selection criteria. A random‐effects model was developed to calculate pooled effect sizes. The DerSimonian‐Laird estimator was used to estimate the variance of the true effect sizes. An interactive dashboard was published to provide transparent analysis and data presentation.

**Results:**

We found no significant effect of URM for BMI, body weight, or percent body fat based on unfiltered pooled effect sizes. Filtered pooled effect size analysis showed a slight adverse effect of URM for total cholesterol and low‐density lipoprotein cholesterol.

**Conclusions:**

Studies did not show an effect of URM on weight gain, obesity, or related metabolic conditions. This may help clinicians when considering the use of URM for patients. Longer studies may be needed for observing obesity development in case the effect of URM on weight gain is small and needs a much longer time to express.

**Study Importance**

**What is already known?**

- Red meat is a nutrient‐rich food that is particularly helpful in obesity management. However, numerous observational studies have linked unprocessed red meat (URM) consumption with obesity and related metabolic disorders.

**What does this review add?**

- Our study of intervention trials did not indicate that URM consumption adversely impacted weight gain, obesity, or related conditions.

**How might these results change the focus of clinical practice?**

- Findings from this study may help clinicians weigh the risks and benefits of recommending URM consumption for patients with obesity concerns.

## INTRODUCTION

Proteins are the building blocks of the body and an essential nutrient for growth, repair, and maintenance. Unprocessed red meat (URM) is among the few foods that provide high‐quality protein, and it is a major source of B vitamins, phosphorus, zinc, and iron. URM forms an important part of the diet and contributes to 19% of total meat consumption in the US diet [1]. However, numerous epidemiological studies have linked URM consumption with obesity, cardiovascular diseases, cancers, and all‐cause mortality. Of the two systematic reviews and meta‐analyses of observational studies, one reported the association of processed and unprocessed meat consumption with obesity, whereas the other did not [2, 3]. Such equivocal results have led to a conundrum on recommending URM consumption in dietary guidelines [4, 5, 6]. Considering the high nutritional value and common and global use of URM in diets [7], it is of public interest to unequivocally understand the risks or benefits of URM consumption.

There seem to be at least two aspects that lend ambiguity to determining the role of URM in obesity and related metabolic disorders. First, many of the studies have been observational, cross‐sectional, or prospective cohort studies [2, 3], which, by definition, can only indicate a correlation but not causation. Second, observational studies usually rely on self‐reported subjective dietary data collected using recalls or food frequency questionnaires [8, 9]. Owing to the well‐documented inaccuracy of self‐reported diet assessment methods, there have been numerous calls to discontinue their use and develop objective methods to assess energy and nutrient intake [10, 11, 12, 13]. Considering these limitations, we undertook a systematic review and meta‐analysis of only parallel‐arm randomized controlled trials (RCTs) or randomized crossover trials (RCOs) to determine what collective conclusion could be drawn regarding the role of URM on obesity or weight gain. As processed red meat and URM are compositionally different, we focused only on the effect of URM consumption.

It should be noted that the intention of this study was not to provide nutritional recommendations regarding continuation or discontinuation of URM in the diet but to objectively inspect available literature for the effect, if any, of URM consumption on obesity, as determined by RCTs.

## METHODS

The systematic review and meta‐analysis were reported according to the Preferred Reporting Items for Systematic Reviews and Meta‐Analyses (PRISMA) guidelines [14]. The systematic review protocol (CRD42020196186) was published in PROSPERO on September 19, 2020, accessible at the following link: www.crd.york.ac.uk/prospero/display_record.php?ID=CRD42020196186.

### Data sources and searches

PubMed, Embase, Web of Science, Scopus, CINAHL Complete, and AGRICOLA databases were searched for English‐language original research articles involving humans that had data regarding URM consumption and obesity or associated factors from inception until August 2020. The detailed search strategies and date searched are documented in Table S1. All studies were imported and duplicates removed from the COVIDENCE website to facilitate the systematic review [15].

### Study selection

URM was defined as URM derived from cows, swine, sheep, goats, and horses, excluding poultry and fish. Studies that reported an association between the exposure of URM consumption and obesity‐related outcomes (i.e., obesity and/or associated factors, such as body mass index [BMI], weight, percent body fat, and serum level of total cholesterol, low‐density lipoprotein [LDL] cholesterol, high‐density lipoprotein [HDL] cholesterol, and triglycerides) were considered. In the primary screening, both observational and interventional studies were included. The final analysis included only RCTs or RCOs in adults with a control group. Of note, our PROSPERO protocol lists children and adult populations of interest. Outcomes only within adults are reported herein owing to the enormity of work for studies involving this population. Findings from book chapters, posters, abstracts, systematic reviews, or articles published in languages other than English were excluded. Additionally, studies that included participants with cancer or any rare diseases were excluded.

**FIGURE 1:**
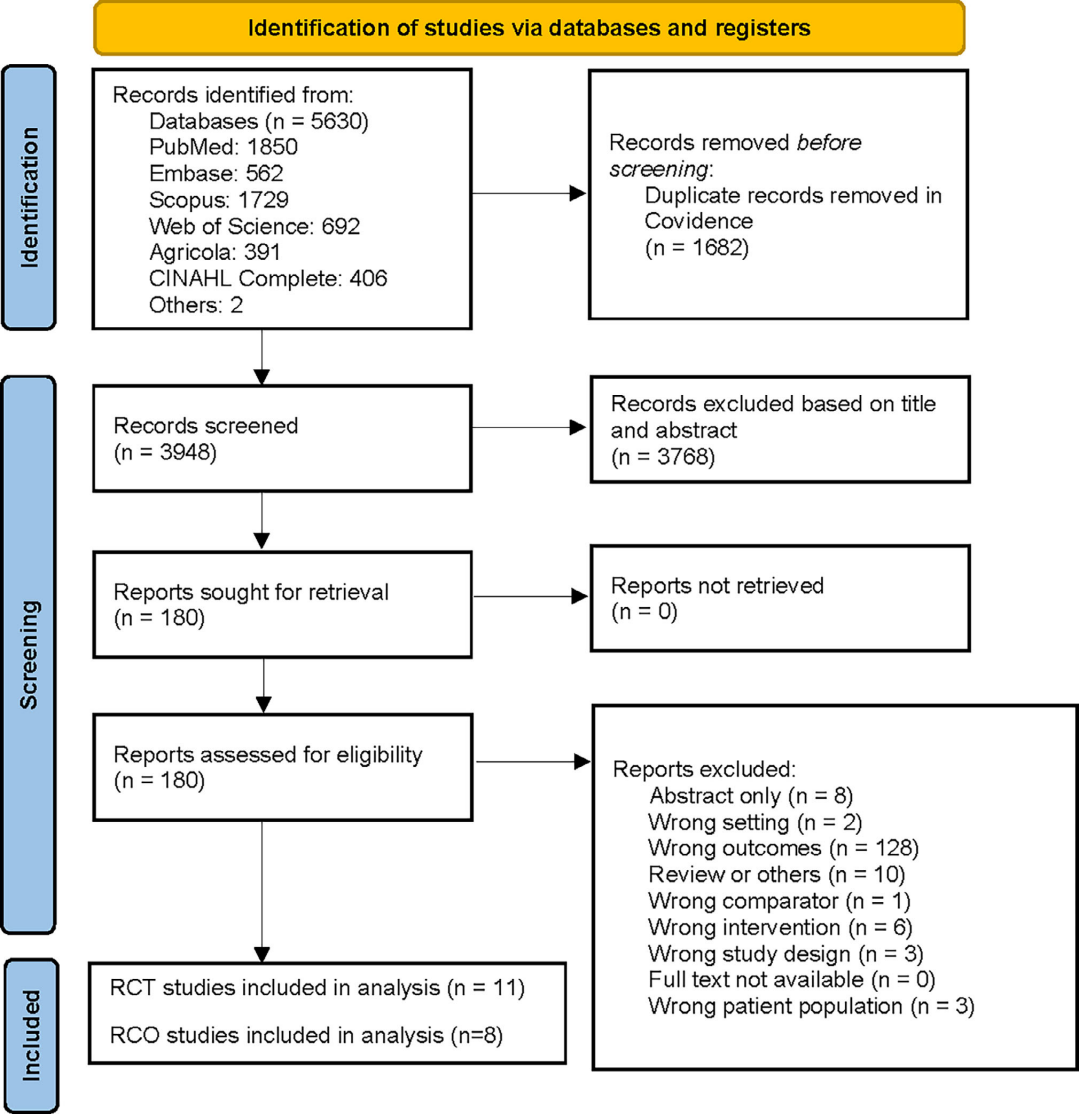
PRISMA (Preferred Reporting Items for Systematic Reviews and Meta‐Analyses) diagram. The process followed for selecting studies. RCO, randomized crossover trial; RCT, randomized controlled trial. [Color figure can be viewed at wileyonlinelibrary.com]

M.A. and M.H. independently screened titles and abstracts of the imported studies in COVIDENCE based on the inclusion and exclusion criteria. N.V.D. adjudicated disagreements between the independent reviewers. The process was anonymous to all reviewers. In the next step, eligible articles were screened through a full text review, followed by detailed assessment of study design, exposure, and outcomes. The selection process of the studies is presented in Figure 1.

### Data extraction and quality assessment

Data were extracted from the eligible articles (Table S2) [16, 17, 18, 19, 20, 21, 22, 23, 24, 25, 26, 27, 28, 29, 30, 31, 32, 33, 34, 35, 36, 37] by each reviewer (M.A. and M.H.), followed by verification. Data for the study characteristics, participant characteristics, intervention details, and outcome details were extracted. A template data collection form is available upon request.

Risks of bias (ROB) of eligible studies was assessed using the Cochrane Collaboration's ROB tool for parallel and crossover design. The ROB tool categorizes the ROB as “low risk,” “some concerns,” and “high risk” across the following five domains: randomization process, deviations from intended interventions, missing outcome data, measurement of the outcome, and selection of the reported result. Answers to all questions and explanations were recorded and compared between reviewers M.A. and M.H. Disagreements were resolved by discussion or adjudicated by a third reviewer, N.V.D. The results for ROB assessment are available in Figure S2.

### Data synthesis and analysis

#### Data preprocessing

All data were prepared for analysis using the software package R (The R Project for Statistical Computing). The R “tidyverse” package was used for all data cleaning and manipulation [38]. For effect size and publication bias analysis, all included studies were divided into two groups, i.e., RCTs and RCOs. The study designs between RCTs and RCOs differ enough to not pool the studies in the analysis. Two studies, i.e., Nadia et al. [39] and Perry et al. [40], were removed from analysis because neither study contained a control group for comparison. Additionally, one study arm from Murphy et al. [33] comparing outcomes under pork versus beef consumption was removed because this comparison does not fit our control‐treatment criteria, as pork and beef are both considered URM.

#### Statistical methods

The “esc” package in R was used to calculate individual effect sizes on standardized mean difference (SMD) for each pairwise comparison from the included studies [41]. Blood lipids were converted from millimoles per liter to milligrams per deciliter by multiplying by 38.67 (LDL cholesterol and total cholesterol) or 88.57 (triglycerides). Owing to differences in reported variability measures, 10 effect sizes were unable to be calculated for metrics from three RCTs. Hill et al. [19] reported triglyceride results as medians and 95% confidence intervals (CI), all measures from Mamo et al. [27] reported means and an undefined measure of variability, and Ziegler et al. [24] reported triglyceride results as medians and interquartile ranges (IQR). Additionally, 10 measures from three RCOs were removed from the analysis. All measures from Smith et al. [35] reported means without a measure of variability, Mateo‐Gallego et al. [32] reported triglyceride and weight results as medians and IQR, and all measures from Maki et al. [31] reported means and nonsymmetric intervals of (−1 SD, +1 SD).

Once all individual effect sizes were calculated, we used a random‐effects model to calculate pooled effect sizes for each outcome [42]. The DerSimonian‐Laird estimator [43] was used to estimate τ^2^, the variance of the true effect sizes. Hartung‐Knapp adjustments to control for uncertainty in our estimate of the between‐study heterogeneity are not used because they would result in wider CI values. Additionally, a fixed‐effects model was used for comparison. We calculated Higgins and Thompson's *I* ^2^ statistic to measure between‐study heterogeneity with reference values of 25%, 50%, and 75%, indicating low, moderate, and substantial heterogeneity, respectively [44].

We conducted an outlier analysis on each pooled effect size using the “dmetar” package in R [45]. This analysis identified individual studies as outliers when the 95% CI of the individual study effect size does not overlap with the 95% CI of the pooled effect size [46]. The pooled effect size was then calculated with outlier studies removed for comparison. The R package “dmetar” was also used to conduct a “leave‐one‐out” meta‐analysis of all pooled effect sizes to test for robustness of the findings [45]. Specifically, the leave‐one‐out process recalculates the pooled effect size after removing each individual study's effect size to determine whether a single study had a large impact on the pooled effect or the heterogeneity of the results [45].

#### Sensitivity analysis

Many of the included studies compared multiple diet treatments, causing single studies to contribute multiple effect sizes to the pooled analysis. In order to correct this, we recalculated all pooled effect sizes twice, filtering studies with multiple treatment arms first for only the arm with the largest negative effect size (URM consumption increases the metric) and then filtering for only the arm with the largest positive effect size (URM decreases the metric). We were unable to calculate filtered pooled effect sizes for percentage body fat, BMI, or weight in the RCOs, owing to these metrics only being reported by one study.

In order to assess the effect of analyzing RCTs and RCOs separately, both unfiltered and filtered pooled effect sizes were also calculated using all studies, regardless of design. Additionally, to account for studies containing multiple treatment arms, a three‐level model was created to calculate pooled effect sizes using the “metafor” package in R [47]. The three‐level model assumes a random‐effects model and has three pooling steps. First, individuals are pooled into treatment arm effect sizes; second, all treatment arms from an individual study are pooled into a study effect size; and third, all study effect sizes are pooled into an overall effect size [46]. In order to calculate the estimated variance of the true effect sizes (τ^2^), the three‐level structure uses the restricted maximum likelihood estimator [48], rather than the DerSimonian‐Laird estimator used in the rest of the analysis. Three‐level models were created for RCTs and RCOs separately and combined.

#### Publication bias

Publication bias is the influence of the findings of an article on the likelihood of that article being published [49]. Publication bias was examined visually using funnel plots, scatterplots of SMD versus standard error (SE), and Egger's test for asymmetry [50]. The R package “metaviz” was used to develop funnel plots, and the “dmetar” package was used to examine asymmetry using Egger's test [46, 50]. We examined publication bias for each analyzed metric separately because scale and sign of the SMD and SE are not consistent among metrics. Asymmetry in funnel plots can indicate publication bias and is detected with an Egger's bias coefficient away from zero [45]. This is because the distribution of the SMD regarding the mean SMD is expected to be normally distributed and therefore symmetric. We also used the trim‐and‐fill method to indicate possible missing studies within the funnel plot that would correct for asymmetry.

Because a single study may compare more than two diet treatments, we considered the pairwise comparisons of these diets while considering the high URM diets as the treatment group consistently. As a result, a single study may appear multiple times in the publication bias analysis. If these results were clustered on one side of the funnel plot, then this could provide a false flag for publication bias. We conducted further analysis to choose a single diet comparison from each individual study by selecting the treatment legs with largest/smallest ratio ([SMD − mean SMD]/SE), thereby choosing the most extreme results from a single diet in an individual study. These choices change the axis of symmetry of the resulting funnel plot (mean SMD). Therefore, we cannot compare the bias values from Egger's test directly and instead focus on the *p* value for each subset.

#### R dashboard

An interactive dashboard (https://danielpballer.github.io/Role_of_Unprocessed_Red_Meat_in_Obesity_and_Related_Factors/) that displays all analysis and code was developed using the “flexdashboard” package in R [51]. This user‐friendly dashboard allows a reader to explore all code, calculations, and data used in this analysis.

## RESULTS

### Effect size

**FIGURE 2:**
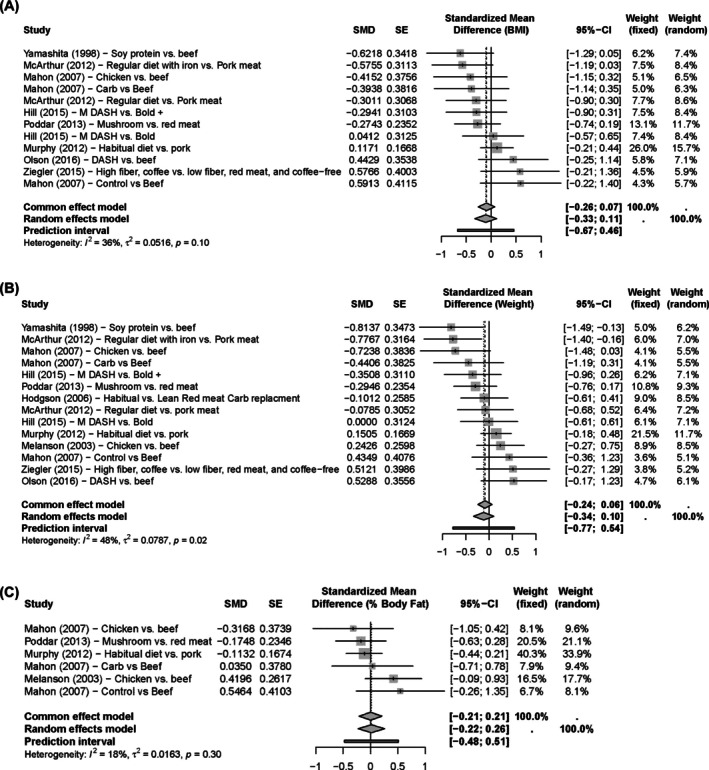
Unfiltered RCT forest plots of effect size and 95% CI for (A) BMI (kilograms per meters squared), (B) weight (kilograms), and (C) fat mass (percentage). All 95% CI for unfiltered pooled effect sizes include 0, indicating no effect of URM consumption on any of the three. SMD estimates and CI to the left of the line of no effect favor non‐URM vs. URM. DASH, Dietary Approaches to Stop Hypertension; RCT, randomized controlled trial; SMD, standardized mean difference; URM, unprocessed red meat.

Figure 2 depicts effect sizes and 95% CI for BMI (Figure 2A), weight (Figure 2B), and percentage body fat (Figure 2C) from the unfiltered analysis of RCTs. All 95% CI for unfiltered pooled effect sizes include 0, indicating no significant effect of URM consumption. Similar results were found for the other outcome measures (see dashboard) for either group, i.e., RCTs and RCOs. In addition, the sensitivity analysis that filtered the largest positive effect size from each study resulted in minor changes to the 95% CI but did not meaningfully change the results. Similarly, filtering for the largest negative effect size from each study had the same result except for LDL cholesterol in the RCTs, which resulted in a 95% CI that did not contain 0 (95% CI: −0.43 to −0.06).

The study by Mahon et al. [18] was identified as an outlier for the unfiltered pooled effect size for total cholesterol, LDL cholesterol, and HDL cholesterol, as well as for total cholesterol when filtering for only the largest negative effect from each study. However, calculating the pooled effect size with this study removed resulted in minimal changes to the 95% CI of the pooled effect size, which did not change the resulting conclusions. For example, when we conducted a leave‐one‐out analysis for Mahon et al. [18] chicken versus beef, the original BMI, percentage body fat, and weight 95% CI were −0.33 to 0.11, −0.22 to 0.26, and −0.34 to 0.10, respectively. Removing this arm from Mahon et al. [18] shifted CI to −0.32 to 0.14, −0.21 to 0.32, and −0.30 to 0.14. A table of the outlier analysis is available in the dashboard.

All unfiltered pooled effect sizes for both RCTs and RCOs had *I* ^2^ statistics less than 50%, except triglycerides for RCTs (54%), indicating low‐to‐moderate heterogeneity. When filtering for the largest negative effect size from each study, the pooled effect for weight in the RCTs had the highest *I* ^2^ statistic of 0.60, indicating moderate heterogeneity. Results of the leave‐one‐out meta‐analysis showed significant overlap with the originally calculated 95% CI for all pooled effect sizes (see dashboard). When filtering for the largest negative effect from each study, Murphy et al. [22] and Wolmarans et al. [37] exhibited sufficient influence to change whether the 95% CI contained 0 for the secondary outcomes of total cholesterol and HDL cholesterol, respectively (see dashboard).

**FIGURE 3:**
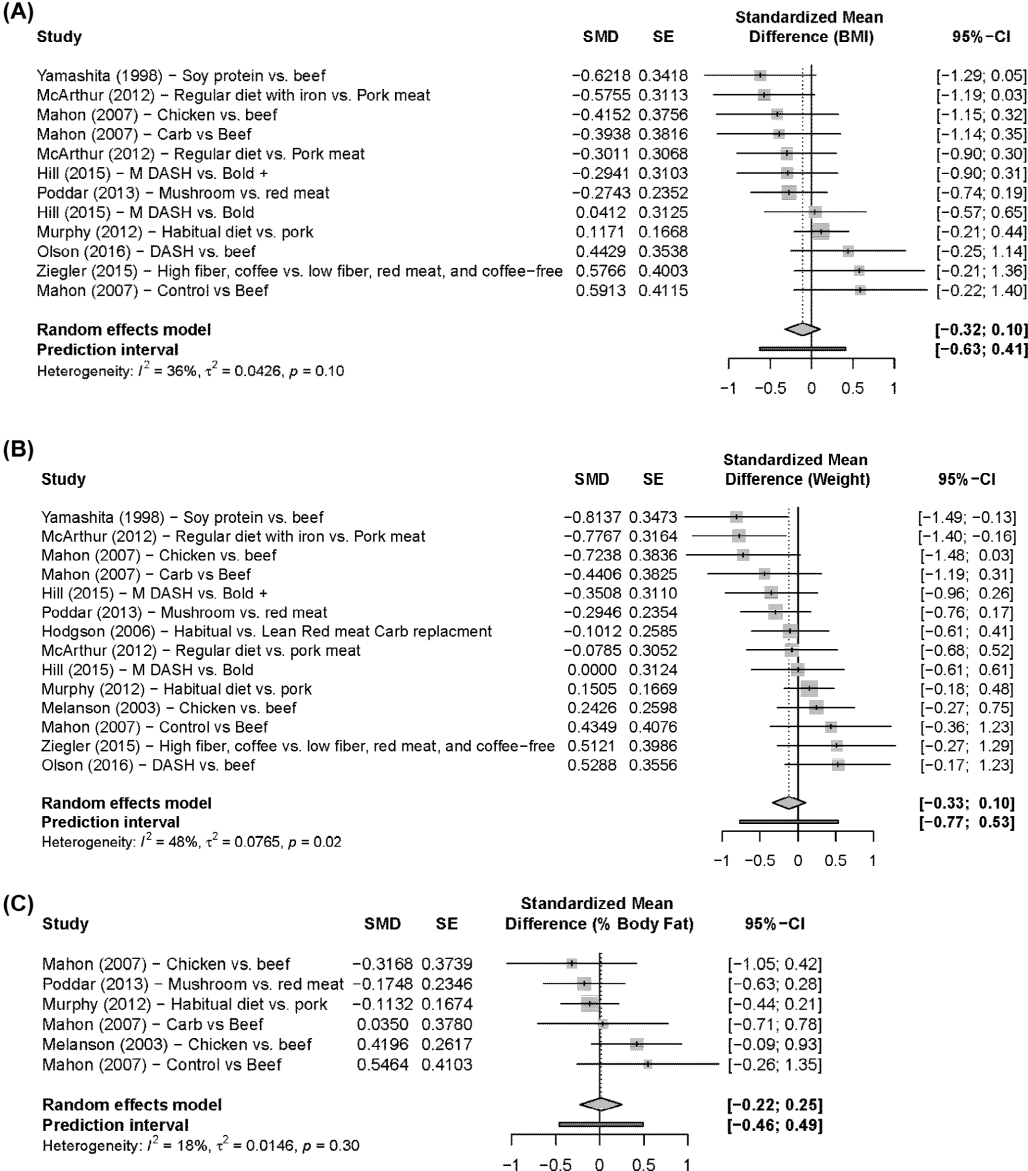
Three‐level model forest plots of effect size and 95% CI for (A) BMI (kilograms per meters squared), (B) weight (kilograms), and (C) fat mass (percentage) outcomes. All 95% CI for multilevel pooled effect sizes include 0, indicating no effect of URM consumption on any of the three. SMD estimates and CI to the left of the line of no effect favor non‐URM vs. URM. Bold, beef in an optimal lean diet; DASH, Dietary Approaches to Stop Hypertension; SMD, standardized mean difference; URM, unprocessed red meat.

Figure 3 depicts effect sizes and 95% CI for BMI (Figure 3A), weight (Figure 3B), and percentage body fat (Figure 3C) outcomes from the three‐level model analysis of RCTs. Three‐level models for all metrics produced the 95% CI for pooled effect sizes that contained 0 when analyzing RCTs and RCOs both separately and combined.

### Publication bias

**FIGURE 4:**
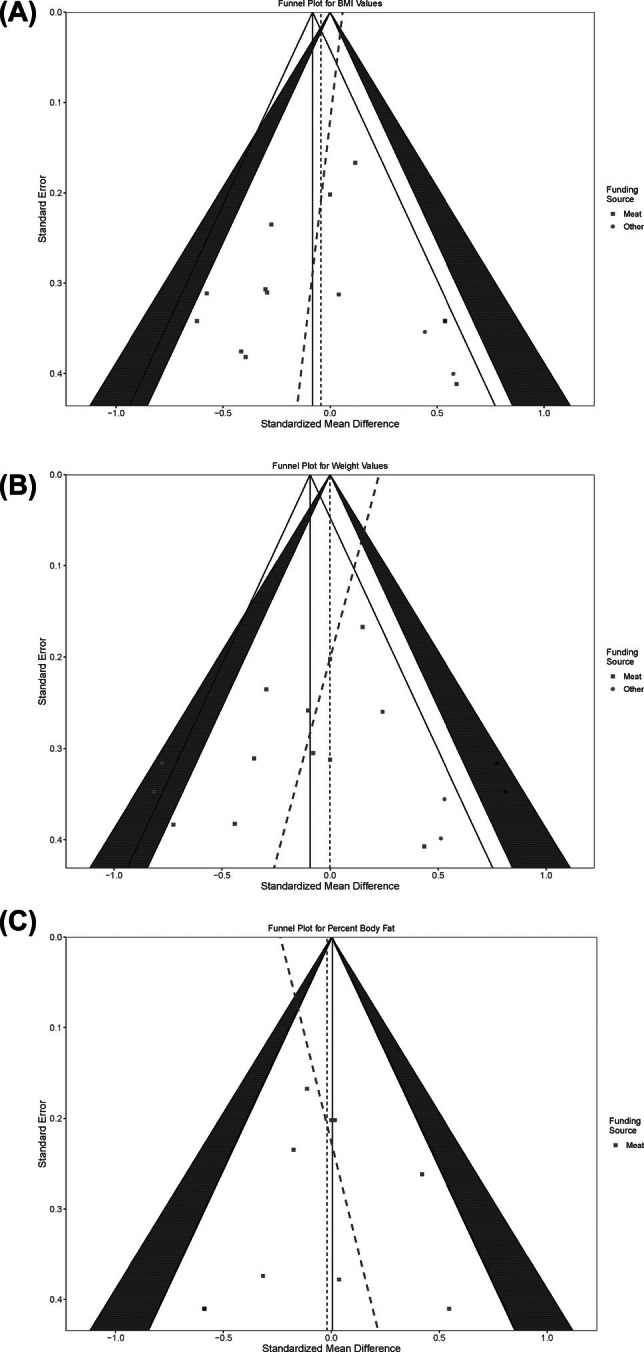
Funnel plots (SMD vs. SE) for unfiltered (A) BMI (kilograms per meters squared), (B) weight (kilograms), and (C) fat mass (percentage) outcomes. Points denoted (Meat, 1) have been imputed using the trim‐and‐fill method to balance the funnel plot. The red dashed line visually represents asymmetry using Egger's test. No significant asymmetry was detected for BMI, weight, or fat mass. SMD, standardized mean difference.

Figure 4 depicts the unfiltered funnel plots (SMD vs. SE) for the three main obesity outcomes of BMI (Figure 4A), weight (Figure 4B), and percentage body fat (Figure 4C). Table S3 includes Egger's test results for all filtered and unfiltered outcomes. The funnel plots are centered around the pooled treatment effect, and the dashed red line shows a visual indication of the asymmetry detected by Egger's test, in which a vertical line represent a perfectly symmetric plot. The funnel plots in Figure 4 show some asymmetry from Egger's test; however, it is not significant, as seen in Table S3. In the unfiltered analysis, we found evidence for publication bias for studies reporting on triglyceride levels (Figure S1). However, this bias was not observed when we selected a single study arm from each study (see dashboard). Similarly, we found publication bias in studies reporting LDL cholesterol and total cholesterol but only when considering the diet comparisons with the maximum values ([SMD − mean SMD]/SE) from each study (Figure S1).

Overall, we observed no significant effect of URM on weight, BMI, percentage body fat, HDL cholesterol, LDL cholesterol, or triglycerides for pooled unfiltered RCTs or RCOs combined or considered separately. Three‐level models for RCTs and RCOs combined or considered separately accounted for multiple treatment arms in some studies and showed no significant effect of URM on all measures. When accounting for multiple treatment arms by filtering studies to keep the treatment arm with the most negative effect size, a small effect of URM was observed on LDL cholesterol (95% CI: −0.43 to −0.06) for RCTs analyzed separately and both LDL (cholesterol 95% CI: −0.30 to −0.06) and total cholesterol (95% CI: −0.27 to −0.01) when analyzing RCTs and RCOs together.

## DISCUSSION

Although meat provides high‐quality protein, a dilemma exists for a clinician regarding the recommendation of meat consumption in dietary guidelines [4, 5, 6]. The guidelines by NutriRECS based on four systematic reviews recommended no changes in the consumption of processed red meat and URM in adults [7]. Other systematic reviews have indicated that the link between URM and disease is weak and of low certainty [52, 53, 54, 55]. On the other hand, there are concerns and controversy regarding the role of URM in health. This discrepancy may be because of the inclusion of observational studies, which are incapable of demonstrating causality, and/or reliance on subjective dietary self‐reports, which tend to be biased and inaccurate diet assessment methods [10, 11, 12, 13]. It is also possible that URM consumption in some situations is linked with a lifestyle of a diet that is higher in energy content. This “guilt by association” may mistakenly lead to conclusions regarding obesogenic properties of URM. We minimized this concern by only focusing on RCTs and RCOs, which, in general, did not show URM as a risk factor for weight gain, development of obesity, or adverse lipid profile.

Herein, we report a very comprehensive analysis, which is made further transparent and accessible with the provision of the accompanying interactive dashboard. We expect that readers will test this user‐friendly dashboard to review how inclusion and exclusion of various studies may impact study variables. However, these findings require careful understanding and interpretation. It is possible that the effect of URM on increasing body weight is too small to be detected in study duration and may need longer‐term intake and/or a higher dose of URM. Moreover, it is difficult to conceive the obesity‐promoting potential of URM, as URM consumption has declined in recent years, whereas the prevalence of obesity, type 2 diabetes, and cardiovascular disease has increased [56, 57, 58, 59]. Also, for comparable amounts of protein, URM, particularly lean URM, has fewer calories per serving compared to other protein sources such as whole dairy, nuts, and beans. It is also possible that hitherto unknown other factors may be involved in free‐living situations, outside of controlled trials. Further investigation may determine whether serving size of URM, fat content, meal timing, frequency of consumption, or accompanying side dishes influence total energy intake through URM. Conversely, if URM does not negatively influence obesity or related metabolic conditions, it may be used strategically for obesity management and other nutritional benefits. Considering that protein‐containing foods can increase satiety and reduce food intake [60], URM, which is a good source of high‐quality protein, may be strategically placed in the daily diet to increase compliance with a weight loss diet and thereby support greater weight loss/maintenance [61, 62].

There are some limitations of this study. First, we did not screen clinical trial registries during the search process. Also, our conclusions are restricted to URM as defined. Some studies used unclear terminology, which prevented us from considering them as a URM study. We note that longer‐term studies may be needed for observing obesity development if the effect of URM on weight gain is small. Finally, this work should be considered as comprehensive up until the search date. This may result in some more recently published literature not being included, which could limit our representation of the current evidence. Finally, all studies had “some concerns” or “high” overall ROB, which warrants caution because it likely represents a limitation in the body of literature included in this review.

This work aims to clarify the relationship between URM consumption and obesity within currently ambivalent literature. Although observational data are equivocal regarding the association of URM with obesity, our study did not indicate that the RCTs and RCOs that tested URM consumption adversely impacted weight gain, obesity, or related metabolic conditions in adults. Our findings can be considered in the generation of evidence‐based guidelines to promote a healthy weight in adulthood and may help clinicians in weighing the merits and drawbacks of the use of URM for patients.

## AUTHOR CONTRIBUTIONS

Conceptualization: Nikhil V. Dhurandhar; study design: Nikhil V. Dhurandhar and Md Akheruzzaman; article screening and processing: Md Akheruzzaman and Marleigh Hefner; statistical analysis: Diana M. Thomas, Shane Clark, and Daniel Baller; writing of original draft: Nikhil V. Dhurandhar, Md Akheruzzaman, Zahra Feizy, Marleigh Hefner, and Daniel Baller; and editing: Marleigh Hefner, Nikhil V. Dhurandhar, Daniel Baller, Diane M. Thomas, and Shane Clark.

## FUNDING INFORMATION

This study received funding from the National Cattlemen's Beef Association (NCBA) Beef Checkoff. Diana M. Thomas was supported by the National Institutes of Health (NIH) Inter 341 Agency Agreement AOD22022001.

## CONFLICT OF INTEREST STATEMENT

Nikhil V. Dhurandhar received grant support from the Beef Checkoff for conducting the systematic review and meta‐analysis. The sponsor did not have any role regarding the study design, data extraction, analysis, or reporting. The other authors declared no conflicts of interest.

## Supporting information


**Data S1.** Supporting Information.

## Data Availability

The data that support the findings of this study are openly available in GitHub at https://danielpballer.github.io/Role_of_Unprocessed_Red_Meat_in_Obesity_and_Related_Factors/.
