# Supplementary material for: Effect of unprocessed red meat on obesity and related factors: A systematic review and meta‐analysis
Source: Obesity (Silver Spring). 2025 Jul 25;33(9):1627–36. doi: 10.1002/oby.24322 (PMC12381610; doi:10.1002/oby.24322)
Supplement: Supplementary file 1 — Data S1. Supporting Information. [file OBY-33-1627-s001.docx]

**Supplemental Materials**

**Figure(s)**

Supplement Figure S1: Funnel plots for blood lipids (pg 2)

Supplement Figure S2: Risk of bias summary (pg 3)

**Table(s)**

Supplement Table S1: Search strategy by database (pg 4)

Supplement Table S2: Characteristics of included studies (pg 7)

Supplement Table S3: Egger’s test for bias and corresponding p-values (pg 16)

**Supplement Figure S1: Funnel plots for blood lipids**


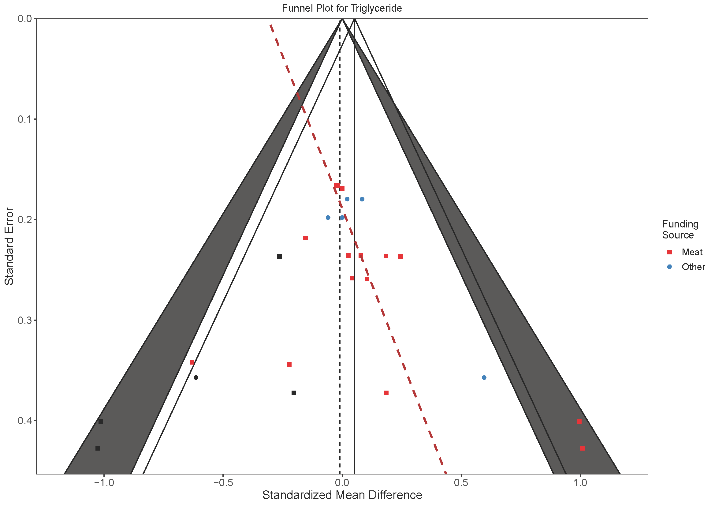

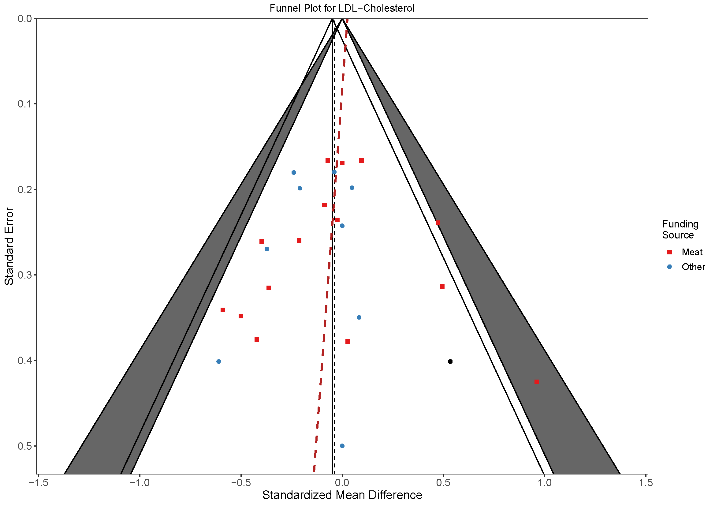

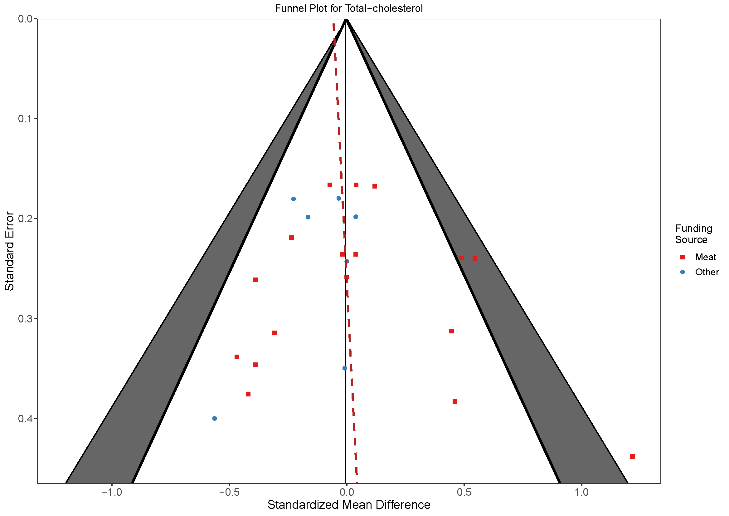


**A.**

**C.**

**B.**

**Supplement Figure S1.** Funnel plots (SMD vs. SE) for unfiltered triglyceride (mg/dL) (Panel A). Funnel plot for max filtered LDL-cholesterol (mg/dL) (Panel B) and total cholesterol (mg/dL) (Panel C). Black nodes are resultant from the trim-and-fill method to show missing data studies. Red dashed line resultant from Egger’s test for asymmetry.

**Supplement Figure S2: Risk of bias summary**


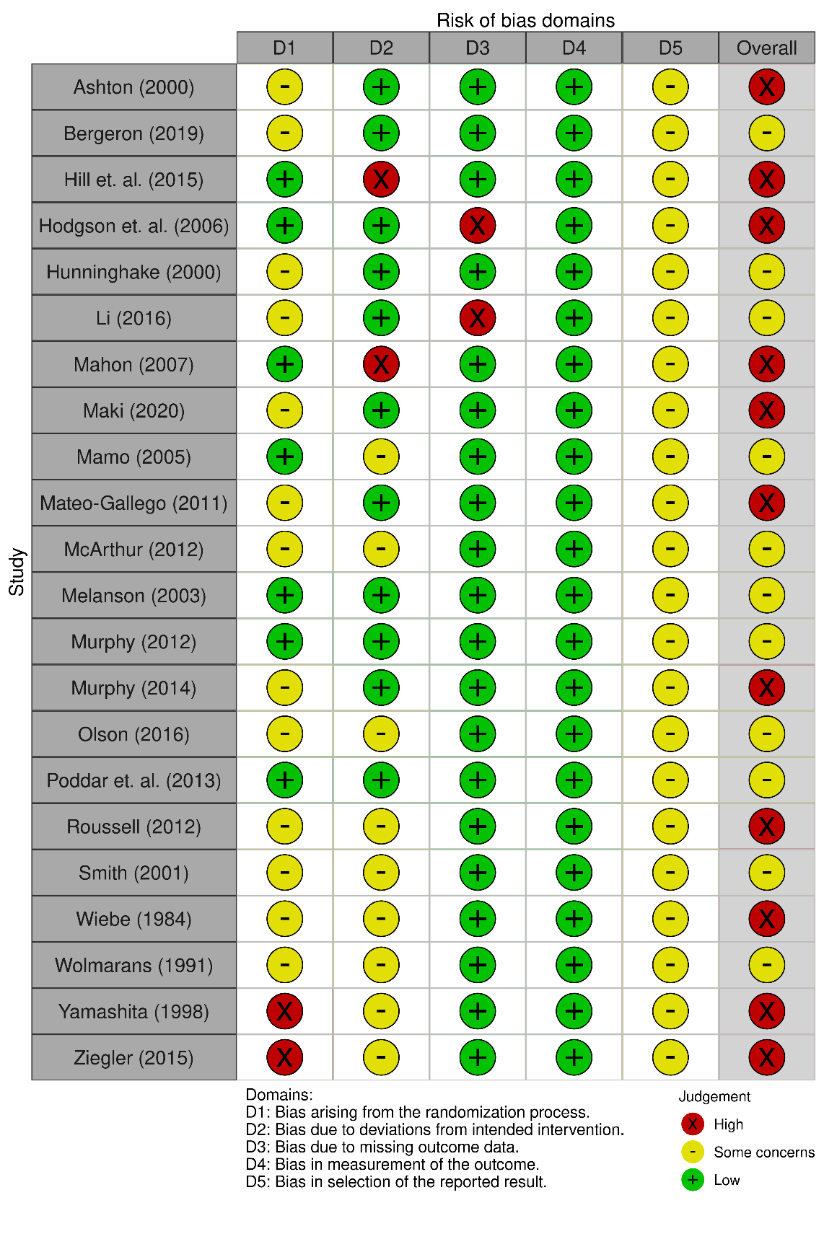


**A.**

**B.**

**
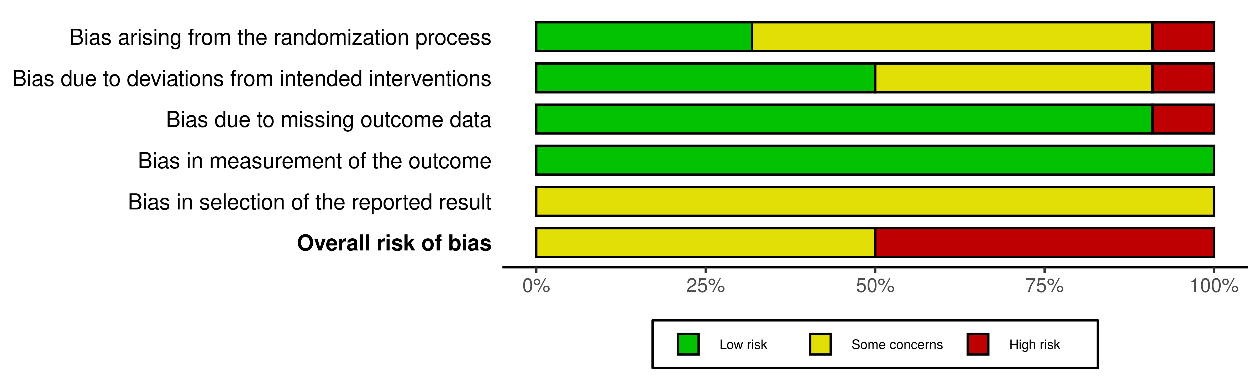
**

**Supplement Figure S2.** Cochrane Collaborations Risk of Bias (RoB 2) traffic light plot (Panel A) and summary plot (Panel B). Studies are presented in alphabetical order by first author last name. The DS (bias arising from period and carryover effects) column specific for crossover studies is not shown but was included as part of the overall result for crossover studies and can be made available upon request. Supplement Figure 2 was created using the *robvis* tool (McGuinness, LA, Higgins, JPT. Risk-of-bias VISualization (robvis): An R package and Shiny web app for visualizing risk-of-bias assessments. Res Syn Meth. 2020; 1- 7. https://doi.org/10.1002/jrsm.1411).

**Supplement Table S1: Search strategy by database**

| **Total: 5430** | | | |
| --- | --- | --- | --- |
| **PubMed on 29 July, 2020 (n=1850)** | | | |
| **No.** | **Search strategy** | | |
| #9 | #7 NOT #8 Filters: Humans, English | | |
| #8 | #3 AND #6 Filters: Books and Documents, Meta-Analysis, Review, Systematic Review | | |
| #7 | #3 AND #6 | | |
| #6 | #4 OR #5 | | |
| #5 | Metabolism[MeSH Terms] OR "Metabolic rate"[All Fields] OR "Resting metabolic rate"[All Fields] OR "BMR"[All Fields] OR "Metabolic rate"[All Fields] OR "Energy consumption"[All Fields] OR "calories"[All Fields] OR "Energy requirement"[All Fields]  OR triglycerides[MeSH Terms] OR triglycerides[All Fields] OR triacylglycerol[All Fields] OR triacylglyceride[All Fields]OR cholesterol[MeSH Terms] OR biomarkers[MeSH Terms] | | |
| #4 | "Adipose Tissue"[MeSH Terms] OR "Body Weights and Measures"[MeSH Terms] "Body Composition"[MeSH Terms]OR "body weight"[MeSH Terms] OR overweight[MeSH Terms] OR obesity[MeSH Terms] OR obesity, morbid[MeSH Terms] OR “body weight changes”[MeSH Terms] OR “weight gain”[MeSH Terms] OR “body mass index”[MeSH Terms] OR bmi[tiab] OR "body mass"[tiab] OR obes*[tiab] OR overweight[tiab] OR weight[tiab] OR "bone Mass"[All fields] OR "Muscle mass"[All fields] OR "Fat free mass"[All fields] OR "lean mass"[All fields] OR "lean body mass"[All fields] OR "body water"[All fields] OR "body water"[All fields] OR "brown fat"[All fields] | | |
| #3 | #1 NOT #2 | | |
| #2 | "Meat Products"[MeSH] OR "Meat Proteins"[MeSH] OR "Poultry"[MeSH] OR "Seafood"[MeSH] | | |
| #1 | Meat[MeSH] OR "Red Meat"[MeSH] | | |
| **Embase on 29 July, 2020 (n=562)** | | | |
| **No.** | **Search strategy** | | |
| #7 | #6 AND 'human'/de AND 'article'/it | | |
| #6 | #1 AND #5 | | |
| #5 | #2 OR #3 OR #4 | | |
| #4 | 'weight'/de OR 'weight gain'/de OR 'obese' OR 'bmi'/de OR 'body mass index'/de | | |
| #3 | 'weight, mass and size'/de OR 'weight, mass and size' | | |
| #2 | 'obesity'/de OR 'obesity' | | |
| #1 | 'red meat'/de | | |
| **Web of science on 29 July, 2020 (n=692)** | | | |
| **No.** | **Search strategy** | | |
| #5 | #3 NOT #4 | | |
| #4 | (TS=("systematic review" OR "review") ) *AND***LANGUAGE:** (English) *AND* **DOCUMENT TYPES:** (Article)  *Indexes=SCI-EXPANDED, SSCI, A&HCI, CPCI-S, CPCI-SSH, BKCI-S, BKCI-SSH, ESCI, CCR-EXPANDED, IC Timespan=All years* | | |
| #3 | #2 AND #1 | | |
| **No. (Cont)** | **Search strategy (Cont)** | | |
| #2 | (TS= ("obesity" OR "obes*" OR "overweight" OR "adipose" OR "adiposity" OR "BMI" OR "body mass index" OR "weight gain") ) *AND***LANGUAGE:** (English) *AND* **DOCUMENT TYPES:** (Article)  *Indexes=SCI-EXPANDED, SSCI, A&HCI, CPCI-S, CPCI-SSH, BKCI-S, BKCI-SSH, ESCI, CCR-EXPANDED, IC Timespan=All years* | | |
| #1 | (TS="red meat") *AND***LANGUAGE:** (English) *AND* **DOCUMENT TYPES:** (Article)  *Indexes=SCI-EXPANDED, SSCI, A&HCI, CPCI-S, CPCI-SSH, BKCI-S, BKCI-SSH, ESCI, CCR-EXPANDED, IC Timespan=All years* | | |
| **Scopus on 21 Aug, 2020 (n=1729)** | | | |
| **No.** | **Search strategy** | | |
| #6 | ( TITLE-ABS-KEY ( "Red meat" ) AND DOCTYPE ( ar ) ) AND ( TITLE-ABS-KEY ( "obesity" OR "obese" OR "overweight" OR "adipose" OR "adiposity" OR "bmi" OR "body" OR "weight" OR "fat" OR "weight gain" ) AND DOCTYPE ( ar ) ) AND ( LIMIT-TO ( EXACTKEYWORD , "Human" ) ) AND ( EXCLUDE ( SUBJAREA , "CHEM" ) ) AND ( LIMIT-TO ( SRCTYPE , "j" ) ) | | |
| #5 | ( TITLE-ABS-KEY ( "Red meat" ) AND DOCTYPE ( ar ) ) AND ( TITLE-ABS-KEY ( "obesity" OR "obese" OR "overweight" OR "adipose" OR "adiposity" OR "bmi" OR "body" OR "weight" OR "fat" OR "weight gain" ) AND DOCTYPE ( ar ) ) AND ( LIMIT-TO ( EXACTKEYWORD , "Human" ) ) AND ( EXCLUDE ( SUBJAREA , "CHEM" ) ) | | |
| #4 | ( TITLE-ABS-KEY ( "Red meat" ) AND DOCTYPE ( ar ) ) AND ( TITLE-ABS-KEY ( "obesity" OR "obese" OR "overweight" OR "adipose" OR "adiposity" OR "bmi" OR "body" OR "weight" OR "fat" OR "weight gain" ) AND DOCTYPE ( ar ) ) AND ( LIMIT-TO ( EXACTKEYWORD , "Human" ) ) | | |
| #3 | ( TITLE-ABS-KEY ( "Red meat" ) AND DOCTYPE ( ar ) ) AND ( TITLE-ABS-KEY ( "obesity" OR "obese" OR "overweight" OR "adipose" OR "adiposity" OR "bmi" OR "body" OR "weight" OR "fat" OR "weight gain" ) AND DOCTYPE ( ar ) ) | | |
| #2 | TITLE-ABS-KEY ( "obesity" OR "obese" OR "overweight" OR "adipose" OR "adiposity" OR "bmi" OR "body" OR "weight" OR "fat" OR "weight gain" ) AND DOCTYPE ( ar ) | | |
| #1 | TITLE-ABS-KEY ( "Red meat" )  AND  DOCTYPE ( ar ) | | |
| **CINAHL Complete (EBSCOhost) on 03 Aug, 2020 (n=406)** | | | |
| **No.** | **Search Strategy** | | **Limiters/Expanders** |
| S11 | S9 AND S10 | | **Limiters** - English Language; Peer Reviewed; Research Article; Human; Language: English; Publication Type: Journal Article  **Expanders** - Apply related words; Apply equivalent subjects  **Search modes** - Boolean/Phrase |
| S10 | S4 OR S5 OR S6 OR S7 | |  |
| S9 | S8 NOT S3 | |  |
| S8 | S1 OR S2 | |  |
| S7 | ( height and weight ) OR waist circumference measurement | |  |
| S6 | Weights and measures | |  |
| S5 | Lipids or cholesterol or triglycerides or lipoprotein | |  |
| S4 | Obese or obesity or overweight | |  |
| S3 | Poultry | |  |
| S2 | MH Meat | |  |
| S1 | Red meat | |  |
| **Agricola (EBSCOhost) on 03 Aug, 2020 (n=391)** | | | |
| **No.** | **Search Strategy** | **Limiters/Expanders** | |
| S9 | S7 AND S8 | **Limiters** - Publication Type: Journal Article; Language: ENGLISH  **Search modes** - Boolean/Phrase | |
| S8 | S1 NOT S2 |  |  |
| S7 | S3 OR S4 OR S5 OR S6 |  |  |
| S6 | ( height and weight ) OR waist circumference measurement |  |  |
| S5 | Weights and measures |  |  |
| S4 | Lipids or cholesterol or triglycerides or lipoprotein |  |  |
| S3 | Obese or obesity or overweight |  |  |
| S2 | Poultry |  |  |
| S1 | Red meat |  |  |

**Supplement Table S2:** **Characteristics of included studies**

| **Author (Year)** | **Study Type** | **Funding Source** | **Trial Registration** | **Participant Characteristics** | **Study Group Definition**  **(Sample Size)** | **Interven-tion Duration (Weeks)** | **Primary Outcome** | **Obesity-Related Measures** |
| --- | --- | --- | --- | --- | --- | --- | --- | --- |
| Yamashita (1998) | RCT | Industry | None | 30-61 years old, women, Overweight/  obese | Control group (n=17): Soy protein (~130g/day)  Intervention group (n=19): Beef (~150g/day)  Meat and soy protein was provided | 16 weeks | Arterial complia-nce, blood pressure, plasma leptin, and plasma lipids | BMI, body weight, TC, LDL-C, HDL-C, and TG |
| Melanson (2003) | RCT | Industry | None | 21-59 years old, women | Control group (n=35): Chicken  Intervention group (n=26): Lean beef  Meat was not provided | 12 weeks | Obesity related | Body weight, % body fat, TC, LDL-C, HDL-C, and TG |
| Mamo (2005)* | RCT | Industry | None | 37-57 years old, adults, dyslipidemic | Control group (n=10): Low protein (14% of energy, source not mentioned)  Intervention group (n=10): High protein (25% of energy from lean red beef, veal, and lamb)  Meat was not provided | 6 weeks | Obesity related | BMI, body weight, % body fat, TC, LDL-C, HDL-C, and TG |
| **Author (Year)** | **Study Type** | **Funding Source** | **Trial Registration** | **Participant Characteristics** | **Study Group Definition**  **(Sample Size)** | **Interven-tion Duration (Weeks)** | **Primary Outcome** | **Obesity-Related Measures** |
| Hodgson (2006) | RCT | Industry | None | >20 years old, adults, hypertensive | Control group (n=31): Regular diet  Intervention group (n=29): Carbohydrate was partially replaced with lean meat (180 or 250 g/day)  Meat was provided | 8 weeks | Systolic blood pressure | Body weight, TC, LDL-C, HDL-C, and TG |
| Mahon (2007) | RCT | Industry and University | None | 50-60 years old, women, menopausal | Control group (n=11 to 15): Habitual, chicken, and carbohydrate (chicken or carbohydrate provided 250 kcal/day, 5 days/week),  Intervention group (n=14): Beef tenderloin (provided 250 kcal/day, 5 days/week)  Meat was provided | 9 weeks | Obesity related | BMI, body weight, % body fat, TC, LDL-C, HDL-C, and TG |
| Murphy (2012) | RCT | Federal/  govt and industry | ACTRN12608000190303 | 18-25 years old, men and women | Control group (n=69 to 72): Habitual diet (<100 g pork per week)  Intervention group (n= 71 or 72): Lean pork (150 g per serving, 5-7 servings/week)  Meat was provided | 24 weeks | Obesity related | BMI, body weight, % body fat, TC, LDL-C, HDL-C, and TG |
| **Author (Year)** | **Study Type** | **Funding Source** | **Trial Registration** | **Participant Characteristics** | **Study Group Definition**  **(Sample Size)** | **Interven-tion Duration (Weeks)** | **Primary Outcome** | **Obesity-Related Measures** |
| McArthur (2012) | RCT | Federal/  govt and industry | None | 18-35 years old, women | Control group (n=22): Regular diet, or w/iron  Intervention group (n=21): Pork meat  Meat was provided | 12 weeks | Serum ferritin | BMI, and body weight |
| Podder (2013) | RCT | Industry | None | 18-65 years old, adults, overweight/  obese | This study compared Mushroom as intervention meat as control, in the current study we flipped the group assignment  Control group (n=36): Mushroom (8 oz a meal, 3 meals/week)  Intervention group (n=37): Meat (90+ % lean beef, 3 meals/week)  Meat/Mushroom was not provided | 24 weeks | Obesity related | BMI, body weight, % body fat, TC, LDL-C, HDL-C, and TG |
| Ziegler (2015) | RCT | Federal/  govt. | NCT01409330 | 18-69 years old, adults, T2D/obese | Control group (n=13): High fiber, free of red meat (poultry allowed), and coffee  Intervention group (n=13): low fiber, red meat (≥150 g/day), and coffee-free  Meat was not provided | 8 weeks | Heart rate variability, inflamm-atory markers, and insulin resistance | BMI, body weight, TC, LDL-C, HDL-C, and TG |
| **Author (Year)** | **Study Type** | **Funding Source** | **Trial Registration** | **Participant Characteristics** | **Study Group Definition**  **(Sample Size)** | **Interven-tion Duration (Weeks)** | **Primary Outcome** | **Obesity-Related Measures** |
| Hill (2015) | RCT | Industry and University | NCT00937638 | 30-60 years old, adults, overweight | 1. M Dash vs. Bold +  2. M Dash vs. Bold  Control group (n=21): M-DASH, ~7% of total animal protein from lean beef  Intervention group (n=21 & 20): BOLD and BOLD+, 62% and 56% of total animal protein sourced from lean beef  Meat was provided | 5 weeks | Obesity related | BMI, body weight, TC, LDL-C, HDL-C, and TG |
| Olson (2016) | RCT | Industry and University | None | 18-65 years old, adults | Control group (n=15): DASH^1^  Intervention group (n=18): Lean beef^2^  Meat was not provided | 12 weeks | Obesity related | BMI, body weight, TC, LDL-C, HDL-C, and TG |
| Li (2016) | RCT | Industry | NCT01005563 | >21 years, adults | The design is crossover within group for various protein percent  Control group (n=17): Lacto-ovo-vegetarian (sources of protein are soy/legume)  Intervention group (n=17): Omnivorous (30% of energy from protein, sources of protein are beef/pork)  Meat was provided | 4 weeks | Obesity related | TC, LDL-C, HDL-C, and TG |
| **Author (Year)** | **Study Type** | **Funding Source** | **Trial Registration** | **Participant Characteristics** | **Study Group Definition**  **(Sample Size)** | **Interven-tion Duration (Weeks)** | **Primary Outcome** | **Obesity-Related Measures** |
| Wiebe (1984) | RCO | Federal/  govt | None | 18-27 years old, men | Control group (n=8): Plant protein diet, soy milk  Intervention group (n=8): Animal protein diet, beef  Meat was provided | 3 weeks  Washout: No | Obesity related | LDL-C and HDL-C |
| Wolmaran (1991) | RCO | Partly industry | None | 22-45 years old, adults | Fish vs. beef  Control group (n=28): Fatty fish (~280 g/day in males, and ~216 g/day in females)  Intervention group (n=28): Red meat (Uncooked beef and mutton, 300 g/day in males, and 225 g/day in females)  Meat was provided | 6 weeks  Washout:  12 weeks | Obesity related | LDL-C and HDL-C |
| Ashton (2000) | RCO | Partly industry | None | 35-62 years old, men | Control group (n=42): Tofu (290 g/day)  Intervention group (n=42): Lean meat (Uncooked, 150 g/day)  Only Tofu was provided | 4 weeks  Washout:  2 weeks | Obesity related | TC, LDL-C, HDL-C, and TG |
| **Author (Year)** | **Study Type** | **Funding Source** | **Trial Registration** | **Participant Characteristics** | **Study Group Definition**  **(Sample Size)** | **Interven-tion Duration (Weeks)** | **Primary Outcome** | **Obesity-Related Measures** |
| Hunningh-ake (2000) | RCO | Industry | None | 18-75 years old, men and women | Control group (n=73): White meat;170 g/day 5-7 days/ week, 80% of which are poultry or fish  Intervention group (n=72): Red meat;170 g/day 5-7 days/ week, 80% of which are lean beef, veal, or pork  Meat was not provided | 36 weeks  Washout:  4 weeks | Obesity related | TC, LDL-C, HDL-C, and TG |
| Smith (2001)* | RCO | Federal/  govt and industry | None | 34-58 years old, men | Low beef vs. high beef  Control group (n=10): Low beef (26 g/day)  Intervention group (n=10): High beef (160 g/day)  Meat was provided | 6 weeks  Washout:  4 weeks | Obesity related | TC, LDL-C, HDL-C, and TG |
| Mateo-Gallego (2011) | RCO | Federal/  govt | None | >18 years old, woman nuns, overweight/  obese | Control group (n=34): Chicken (125 g 3 days/week)  Intervention group (n=34): Lamb (125 g 3 days/week)  Meat was provided | 4 weeks  Washout: No | Obesity related | Body weight, TC, LDL-C, HDL-C, and TG |
| **Author (Year)** | **Study Type** | **Funding Source** | **Trial Registration** | **Participant Characteristics** | **Study Group Definition**  **(Sample Size)** | **Interven-tion Duration (Weeks)** | **Primary Outcome** | **Obesity-Related Measures** |
| Roussell (2012) | RCO | Industry | NCT00937898 | 30-65 years old, men and women | Control group (n=36): Healthy American Diet, HAD (**20 g lean beef/day**) or DASH (**28 g lean beef/day**)  Intervention group (n=36): BOLD^3^ (113 g lean beef/day) or BOLD+^3^ (153 g lean beef/day)  Comparison:  1. HAD vs. BOLD+  2. HAD vs. BOLD  3. DASH vs. BOLD+  4. DASH vs. BOLD  Meat was provided | 5 weeks  Washout:  1 week | Obesity related | TC, LDL-C, HDL-C, and TG |
| Murphy (2014) | RCO | Federal/  govt and industry | ACTRN12610000612011 | Middle aged, men and women | Control group (n=49): Chicken (150 g per servings, men 7 servings/week, and women 5 servings/week)  Intervention group (n=49): Beef (150 g per servings, men 7 servings/week, and women 5 servings/week) or Pork (140 g per servings, men 7 servings/week, and women 5 servings/week)  Comparison:  1. Chicken vs. Beef  2. Chicken vs. Pork  3. Pork vs. Beef  Meat was provided | 12 weeks  Washout: No | Metabolic syndrome criteria | BMI, body weight, and % body fat |
| **Author (Year)** | **Study Type** | **Funding Source** | **Trial Registration** | **Participant Characteristics** | **Study Group Definition**  **(Sample Size)** | **Interven-tion Duration (Weeks)** | **Primary Outcome** | **Obesity-Related Measures** |
| Bergeron (2019) | RCO | Federal/  govt | NCT01427855 | 21-65 years old, adults | Control group (n=51 or 62): Low-SF^4^ Arm, White meat (11% of total energy from poultry) or Nonmeat (16% of total energy from vegetable protein); High-SF Arm, White meat (11.5% of total energy from poultry) or Nonmeat (15.4% of total energy from vegetable protein)  Intervention group (n=51 or 62): Low-SF Arm, Red meat (12.5% of total energy from beef); High-SF Arm, Red meat (11.5% of total energy from beef)  Comparison:  1. White meat vs. red meat (Low-SF ARM)  2. Non-meat vs. red meat (Low-SF ARM)  3. White meat vs. red meat (High-SF ARM)  4. Non-meat vs. red meat (High-SF ARM)  Meat was provided | 4 weeks  Washout:  2-7 weeks | Obesity related | TC, LDL-C, HDL-C, and TG |
| **Author (Year)** | **Study Type** | **Funding Source** | **Trial Registration** | **Participant Characteristics** | **Study Group Definition**  **(Sample Size)** | **Interven-tion Duration (Weeks)** | **Primary Outcome** | **Obesity-Related Measures** |
| Maki (2020)* | RCO | Industry | NCT03202680 | 18-74 years old, adults, overweight/  obese | Low meat vs. high meat  Control group (n=33): USDA-CON (<40 g/day red meat)  Intervention group (n=33): USDA-LB (150 g/day lean beef)  Meat was provided | 4 weeks  Washout:  ≥2 weeks | Obesity related | TC, LDL-C, HDL-C, and TG |
| Nadia (2008)* | No control interv-ention | Industry |  | >30 years old, adults |  |  | Obesity related | TC and TG |
| Perry (2020)* | No control interv-ention | Federal/  govt. and industry |  | >65 years old, men and women |  |  | Obesity related | BMI, body weight, and % body fat |
| ^1^DASH (Dietary Approach to Stop Hypertension) diet provided 15%, 55%, and 30% of energy from protein, carbohydrate, and fat, respectively  ^2^Diet provided 30%, 40%, and 30% of energy from protein (1/2 from lean beef), carbohydrate, and fat, respectively  ^3^BOLD (Beef in optimal lean diet); BOLD+ (Beef in optimal lean diet Plus additional protein)  ^4^SF: Saturated Fat Arm  *Articles which met inclusion criteria but were not included in final analysis due to reporting unusable measures of variability (Mamo 2005; Smith 2001; Maki 2020) or had no control group (Nadia 2008; Perry 2020).  Abbreviations: RCT (randomized controlled trial); RCO (randomized crossover trial); BMI (body mass index); TC (total cholesterol), LDL-C (low-density lipoprotein cholesterol), HDL-C (high-density lipoprotein cholesterol), and TG (triglyceride) | | | | | | | | |

**Supplement Table S3: Egger’s test for bias and corresponding p-values**

| **Egger’s Bias Test Result** | | | | | | |  |
| --- | --- | --- | --- | --- | --- | --- | --- |
|  | **Unfiltered** | | **Min Filtered** | | **Max Filtered** | |  |
| **Metric** | **Bias** | **p-value** | **Bias** | **p-value** | **Bias** | **p-value** |  |
| Weight Values | 1.448 | 0.442 | 2.323 | 0.394 | 0.399 | 0.880 |  |
| Total-cholesterol | -0.226 | 0.800 | 1.596 | 0.021 | 0.147 | 0.910 |  |
| LDL-Cholesterol | 0.286 | 0.714 | 1.233 | 0.044 | 0.494 | 0.649 |  |
| HDL-Cholesterol | -0.094 | 0.901 | 0.516 | 0.565 | -0.245 | 0.823 |  |
| Triglyceride | -1.597 | 0.048 | -0.199 | 0.844 | -1.007 | 0.440 |  |
| BMI Values | 0.709 | 0.703 | NA | NA | NA | NA |  |
| Percent Body Fat | NA | NA | NA | NA | NA | NA |  |
|  |  |  |  |  |  |  |  |
| *NA indicates metrics with fewer than 10 results,* *which are excluded for lack of accuracy.* | | | | | | | |
